# Iterative qualitative approach to establishing content validation of a patient-reported outcome measure for arm lymphedema: the LYMPH-Q Upper Extremity Module

**DOI:** 10.1186/s41687-024-00701-3

**Published:** 2024-06-26

**Authors:** Manraj N. Kaur, Sylvie D. Cornacchi, Elena Tsangaris, Lotte Poulsen, Louise M. Beelen, Louise Bordeleau, Toni Zhong, Mads Gustaf Jorgensen, Jens Ahm Sorensen, Babak Mehrara, Joseph Dayan, Andrea L. Pusic, Anne F. Klassen

**Affiliations:** 1 Department of Surgery, Brigham and Women’s Hospital, Harvard Medical School, 75 Francis Street, Boston, MA 02115 USA; 2 Department of Pediatrics, 3N27, McMaster University, 1280 Main Street West, Hamilton, ON L8N 3Z5 Canada; 3 Modus Outcomes, CIC 1 Broadway, 14th Floor, Cambridge, MA 02142 USA; 4 Research Unit for Plastic Surgery, Odense University Hospital, J.B. Winsløws Vej 4, Odense C, DK-5000 Denmark; 5 Department of Plastic and Reconstructive Surgery, Erasmus MC University Medical Center, Rotterdam, The Netherlands; 6 Department of Oncology, Juranvinski Cancer Center, 699 Concession Street, Hamilton, ON L8V 5C2 Canada; 7 Division of Plastic and Reconstructive Surgery, University Health Network, Toronto General Hospital, 8NU-871, 200 Elizabeth Street, Toronto, ON M5G 2C4 Canada; 8 Memorial Sloan-Kettering Cancer Centre, 1275 York Avenue, New York, NY 10065 USA; 9 Department of Surgery, Brigham and Women’s Hospital, Harvard Medical School, 75 Francis Street, Boston, MA 02115 USA

**Keywords:** Lymphedema, Patient-reported outcomes, Patient-reported outcome measure, Breast cancer-related lymphedema, Arm swelling

## Abstract

**Background:**

Breast cancer-related lymphedema (BRCL) is one of the most common causes of upper extremity (UE) lymphedema in developed nations and substantially impacts health-related quality of life. To advance our understanding of the epidemiology and treatment of BRCL, rigorously developed and validated patient-reported outcome measures (PROMs) are needed. This study aimed to demonstrate the iterative content validity of a modular UE lymphedema-specific PROM called the LYMPH-Q UE module.

**Methods:**

A multi-step iterative qualitative approach was used. Semi-structured interview data from in-depth qualitative interviews with adult women (18 years and older) with BCRL were used to develop the first set of the LYMPH-Q UE scales. The content validity of these scales was demonstrated with patient and clinician feedback. Over the course of cognitive debriefing interviews, additional concepts of lymphedema worry and impact on work were identified as missing from the LYMPH-Q UE module. Subsequently, two new qualitative studies (a focus group and in-depth concept elicitation interviews with patients) were conducted, and two new scales were developed to measure lymphedema worry and impact on work life and their content validity was demonstrated.

**Results:**

Qualitative data from in-depth and cognitive interviews with 15 (age 40–74 years) and 16 (age 38–74 years) women with BRCL, respectively, and feedback from 12 clinical experts, were used to develop and demonstrate the content validity of six LYMPH-Q UE scales measuring symptoms, function, appearance, psychological, information, and arm sleeve. Additionally, data from in-depth interviews with 12 (age 35–72 years) women with UE lymphedema and four focus groups (n = 16 women; age 35–74 years) was used to develop and assess the content validity of two new LYMPH-Q UE scales measuring lymphedema worry and impact on work life. The content validity of the previously established six scales was also demonstrated in these subsequent qualitative studies.

**Conclusion:**

The LYMPH-Q UE is a modular PROM developed using international guidelines for PROM development and can be used in clinical practice, research, and quality improvement to enhance patient-centered shared decision-making. This study’s innovative and iterative approach to content validation demonstrates that the LYMPH-Q UE is a comprehensive measure that includes important concepts relevant to patients with UE lymphedema.

**Supplementary Information:**

The online version contains supplementary material available at 10.1186/s41687-024-00701-3.

## Introduction

Breast cancer-related lymphedema (BCRL), which results from oncologic treatment-related disruption to the lymphatic system, is one of the most common causes of upper extremity lymphedema in developed nations. A recent meta-analysis estimated that one in five breast cancer survivors will develop BCRL, and the risk of developing lymphedema increases for up to 2 years after the cancer diagnosis or surgery [1]. BCRL manifests as upper extremity swelling, heaviness, pain, tightness, skin changes, and reduced arm mobility. These symptoms and function-related impairments are often progressive and associated with a range of physical, emotional, and social sequelae impacting women’s overall health-related quality of life (HRQL). The management of BCRL requires a multidisciplinary approach and may consist of non-surgical (e.g., compression, manual lymphatic drainage, exercise) and surgical (e.g., lymph node transplant, lymphovenous bypass, liposuction) interventions [2]. Accurate and timely assessment of the presence and severity of lymphedema is critical to preventing the worsening of BCRL.

BCRL is assessed using patient history, review of risk factors, clinical examination including observation, palpation of the arm for pitting edema, stemmer sign, arm circumference, diagnostic tests such as lymphoscintigraphy, Indocyanine green (IGC)—enhanced near-infrared fluorescence, and patient-reported outcomes [2–4]. While clinical examination and diagnostic tests provide valuable information, they do not capture the multidimensional HRQL impact of BCRL. Patient-reported outcome measures (PROMs) are instruments designed to capture the range of HRQL concepts that can be best known by asking patients without any interpretation by a clinician or anyone else. Recent systematic reviews [5, 6] have identified several PROMs explicitly developed for upper extremity lymphedema, including the Lymphedema Quality of Life Questionnaire (LYMQOL) [7], the Upper Limb Lymphedema 27 Questionnaire (ULL-27) [8], the Lymphedema Functioning, Disability and Health Questionnaire (Lymph-ICF) [9, 10], and the Lymphedema Symptom Intensity and Distress Survey – Arm (LSIDS-A) [11]. A common limitation that underpins most of these PROMs is that they were developed with minimal patient input and did not follow established guidelines [12, 13]. Further, they do not capture the full range of HRQL issues that matter to women with BCRL [5, 6].

**Fig. 1:**
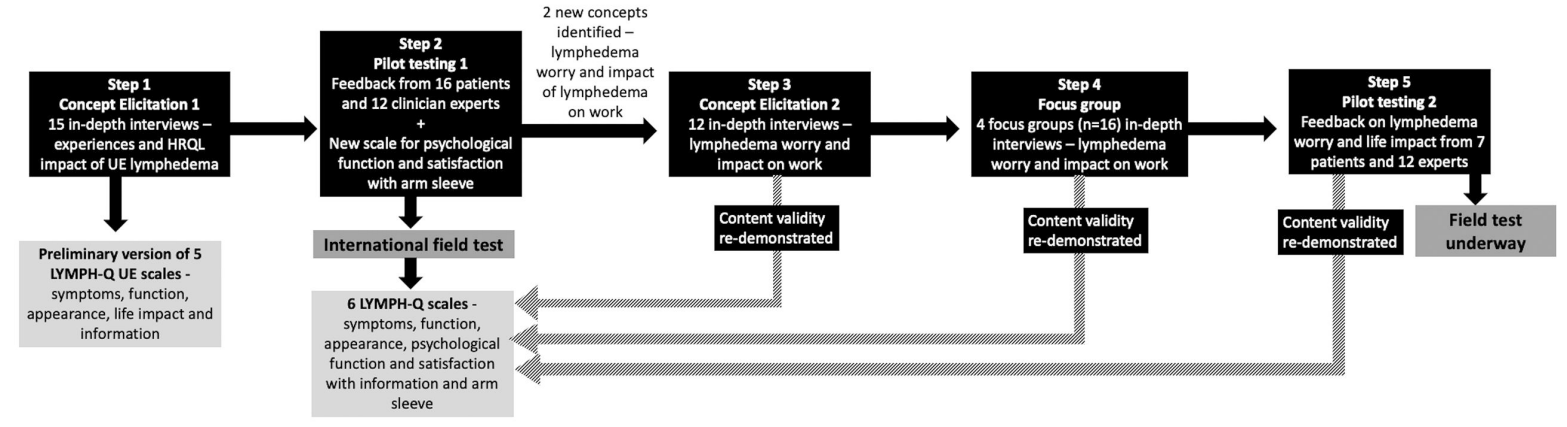
Conceptual framework of the LYMPH-Q Upper Extremity Module

To address these gaps, our team developed an upper-extremity lymphedema-specific PROM called the LYMPH-Q Upper Extremity (LYMPH-Q UE) which has been previously published [14]. This modular, concept-driven PROM was developed using extensive patient input, followed best practice guidelines for PROM development [12, 13, 15, 16], and utilized a modern psychometric approach (i.e., Rasch Measurement Theory (RMT) analysis) [17, 18]. This paper describes the multi-step iterative qualitative approach to developing the LYMPH-Q UE conceptual framework (see Fig. 1) and set of independently functioning scales.

## Methods

The ethics approval for the study was obtained from the Hamilton Integrated Research Ethics Board (Juranvinski Cancer Center (JCC), Hamilton, Ontario, Canada) and from the human research ethics boards of Toronto General Hospital (TGH; Toronto, Ontario, Canada), Memorial Sloan Kettering Cancer Center (MSK; New York, New York, U.S.) and Brigham and Women’s Hospital (BWH; Boston, Massachusetts, U.S.). In Denmark (DK), the study was reported to and approved by the Region of Southern Denmark and was included on the list of Health Research for data protection safety. Written and verbal consent was obtained from all participants before the interviews. Study participants in Canada and the U.S. were given a $50 (CAD, USD) gift card to thank them for participating.

### Approach

We used the health services research-specific qualitative approach called interpretive description [19] for this study. The development and content validation of the LYMPH-Q UE module occurred in a multi-step and iterative manner (Fig. 1). Each of these steps is described below.

#### Step 1: Concept Elicitation 1—for initial LYMPH-Q UE scales

A series of in-depth concept elicitation interviews were conducted between January 2017 and June 2018 with a maximum variation sample of English-speaking, adult (18 years or older) women with breast cancer who varied by age, stage, and treatment of breast cancer. The primary objective of these interviews was to create a Utility module for the BREAST-Q, and the detailed protocol for the BREAST-Q Utility module development study is published elsewhere [20, 21]. The interviewer contacted the participant to explain the study procedures, obtained consent and conducted the interview (by telephone or in person). During the interviews, in-depth information on the impact of diagnosis and treatment of breast cancer on participants’ HRQL was elicited—(see Supplementary Material Files, Appendix 1 for interview guide). Interviews continued until data saturation was reached. Data were coded in Microsoft Office Word using a line-by-line approach and transferred to Excel using Doctools© for further refinement using constant comparison. An item pool was developed from the codes for use in scale development.

The data analysis led to the development of the BREAST-Q Utility module and identified gaps in the BREAST-Q content. One of the gaps was the limited coverage of concepts relevant to arm lymphedema. The data from the subset of participants with BCRL in this sample who provided rich information about their BCRL-related experiences were used to draft the LYMPH-Q UE. The LYMPH-Q UE (version 1) consisted of five upper extremity lymphedema-specific scales that measured symptoms, function, appearance, life impact, and information. For each scale, the instructions, a time frame for answering, and a set of response options were drafted.

#### Step 2: Pilot Testing 1—for initial LYMPHQ UE scales

A series of cognitive debriefing interviews were conducted with English-speaking women with BCRL from JCC, MSK, and DK to refine and establish the content validity of five LYMPH-Q UE scales. The “think aloud” technique [22] was used, and patients were asked to comment on the comprehensibility of each component of the scale (i.e., instructions, timeframe, response options, and items) and the comprehensiveness and relevance of the items and the scale [15, 16]. At the end of each scale, participants were asked to describe any concepts they thought were missing.

The cognitive debriefing interviews took place in three rounds, with changes made to the LYMPH-Q UE between rounds. Interviews in Rounds 1 and 3 were in English. These interviews were recorded, transcribed, and analyzed line-by-line. Interviews in Round 2 were in Danish [23] and were not recorded due to the need for translation before coding. Instead, for these interviews, the qualitative interviewer made detailed notes, which were reviewed by the study team and used to make revisions. Feedback was sought from a group of BCRL experts known to the investigators after Round 2. A research team member sent an email invitation with a copy of the LYMPH-Q UE scales. Experts were asked to provide written feedback via email and to add missing concepts. One reminder email was sent after 1-week. Patient and expert input was used to refine the LYMPH-Q UE and demonstrate content validity.

#### Step 3: Concept Elicitation 2—for lymphedema worry and impact on work concepts

The expert consults identified the need for two additional scales measuring lymphedema worry and impact on work. As the Step 1 concept elicitation was not targeted to BCRL, we did not have saturation for these two concepts. Consequently, a new series of qualitative interviews were conducted with English-speaking women with BCRL recruited from JCC between July and December 2020 to probe these concepts. The interview guide for this study is included in the Supplementary Material Files (see Appendix 2). The recruitment followed the procedures described in Step 1. All the interviews were conducted over the phone due to the COVID-19 pandemic. Interviews were audio-recorded, transcribed, and coded using the approach described in Step 1.

#### Step 4: Focus Groups—content validity for all HRQL concepts

As part of a separate study to understand patient priorities and preferences for upper extremity lymphedema research [24], focus group interviews were conducted over secure, encrypted and institutionally approved Zoom video-conferencing platform between May 2021 and November 2021, with English-speaking women with BCRL recruited from TGH and JCC. These interviews included women with UE lymphedema who were managed conservatively or surgically or had had surgery for lymphedema. The recruitment followed the procedures described in Step 1. A section of the focus group sessions had participants describe the impact of UE lymphedema on their HRQL regarding physical symptoms, social life, work, appearance, emotional distress, and sexual well-being (Supplementary Material Files, Appendix 3 for the interview guide). Focus group sessions were audio-recorded, transcribed, and analyzed using the line-by-line approach described in Step 1. The HRQL data from the focus group sessions was mapped to the content of the 6 LYMPH-Q UE scales to provide evidence of content validity and support the development of two new scales for lymphedema, lymphedema worry and impact on work.

#### Step 5: Pilot Testing 2—for two new LYMPH-Q UE scales (lymphedema worry and impact on work)

The methodology described in Step 2 (Pilot testing 1) was followed. Cognitive debriefing interviews were conducted in two rounds with English-speaking women with BCRL (managed with or without surgery) recruited from the focus group (Step 4) sample between February and March 2022. Expert feedback was sought between the rounds using the methods described in Step 2. Patient and expert feedback was used to refine the scales and establish content validity.

### Rigor

The interviews were transcribed by a professional, third-party company for all the steps. The data collection and analysis occurred concurrently such that new concepts were added to the interview guide iteratively. All interviews were independently coded by two coders (Steps 1 and 3) or coded by one coder and checked by another (Steps 2 and 4). The coders regularly met to review the codebook and reach consensus on coding discrepancies. The codes and the evolving conceptual framework were reviewed in research team meetings.

## Results

### Step 1: Concept Elicitation 1—for initial LYMPH-Q UE scales

**Fig. 2:**
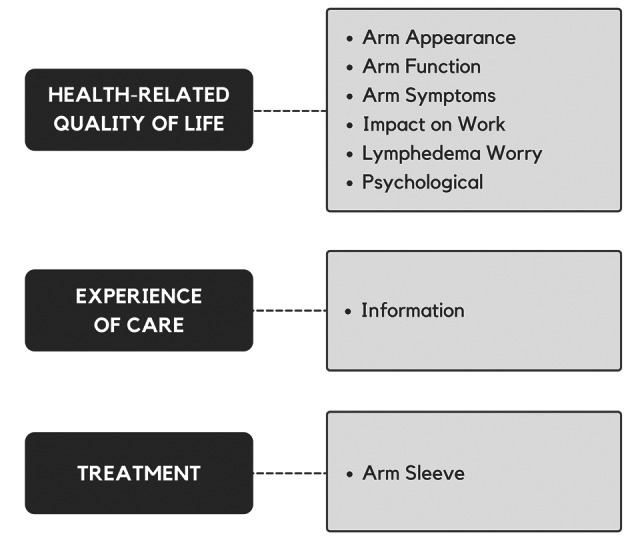
Iterative concept elicitation and content validation of the LYMPH-Q Upper Extremity Scales

Qualitative interviews were performed with 57 patients in the larger BREAST-Q-Utility module study. Data from 15 participants with confirmed or suspected BCRL (i.e., patients in whom chronicity of arm lymphedema has not been established or in whom arm swelling or other symptoms of BCRL are being monitored) was used to develop the LYMPH-Q UE scales. These participants were aged between 40 and 74 years, mainly White (n = 13) and married (n = 10). Most had a mastectomy (n = 10) and a history of having combination treatment with chemotherapy, radiotherapy, or endocrine therapy (n = 7). Table 1 shows the sample characteristics. Analysis of the qualitative data for this subset of participants led to the development of a conceptual framework that included top-level domains with two or more of the following major themes: arm appearance (body image, characteristics, clothing), physical (function, symptoms), psychological (distress, impact), social (support, function, relationships) and experience of care (information) and treatment (sleeve) (Fig. 2).

**Table 1:** Characteristics of participants

		Step 1 Concept elicitation 1 n = 15	Step 2 Pilot testing 1 n = 16	Step 3 Concept elicitation 2 n = 12	Step 4 Focus group n = 16	Step 5 Pilot testing 2 n = 7
Country	Canada	8	1	12	16	7
	USA	7	5	0	0	0
	Denmark	0	10	0	0	0
Age	<39	0	1	2	1	1
	40–49	5	3	3	3	2
	50–59	5	2	3	8	3
	60–69	3	4	3	3	1
	≥70	2	4	1	1	0
Race	White	13	–	12	14	6
	Other	2	–	0	2	1
BMI	Underweight (<18.5)	1	–	0	0	0
	Normal weight (18.5–24.9)	4	–	1	5	3
	Overweight (25–29.9)	6	–	4	4	2
	Obese (≥30)	4	–	7	5	2
	Missing	0		0	1	0
Marital status	Married	10	11	9	10	4
	Common law	2	1	0	1	1
	Separate/divorced	1	1	2	4	2
	Single	1	1	0	1	0
	Widowed	1	1	1	0	0
Highest education	<High school	0	–	1	0	0
	High school	5	–	2	4	0
	College, trade or university	7	–	7	9	3
	Masters or Doctoral degree	3	–	2	3	4
Work	Working full-time	8	–	6	7	5
	Self-employed	1	–	1	0	1
	Causal work	1	–	0	0	1
	Retired	2	–	3	2	0
	Not working	3	–	2	7	0
Household income	<25K	1	–	0	1	0
	25K–50K	2	–	1	1	1
	50K–75K	3	–	4	2	0
	>75K	6	–	7	8	5
	Prefer to not say	3	–	0	4	1
Time since diagnosis	<1 year	2	–	0	0	0
	1–5 years	10	–	7	4	1
	>5 years	3	–	5	11	6
Cancer stage	1	3	–	0	0	0
	2	6	–	8	2	2
	3	6	–	4	8	3
	4	0	–	0	3	1
	Not sure	0	–	0	2	1
	Not applicable	0	–	0	1	0
Surgery type	Mastectomy	12	11	10	8	4
	Lumpectomy	3	4	2	6	3
	Other^a^	0	0	0	2	0
Breast reconstruction	Yes	8	–	0	–	–
	No	7	–	12	–	–

Data for one U.S.A cognitive debriefing interview participant is missing

^a^Focus groups included one participant with melanoma and one participant with ovarian cancer

The item pool was used to develop five preliminary scales for the LYMPH-Q UE Module with 57 items: symptoms (n = 18), function (n = 7), appearance (n = 11), life impact (n = 9), and information (n = 12). Each scale was assigned instructions, a time frame for responding, and four response options that measured severity (symptoms, life impact), bother (appearance), difficulty (function), or satisfaction (information). Table 1 in Appendix 4 (Supplementary Material Files) shows illustrative quotes from the patients for these concepts.

### Step 2: Pilot Testing 1—for initial LYMPH-Q UE scales

Sixteen women with BCRL took part in a cognitive debriefing interview; Round 1 included two U.S. participants, Round 2 included 10 Danish participants, and Round 3 included one Canadian and three U.S. participants. The participants were aged between 38 and 74 years, mainly White (n = 16) and married (n = 11). Most participants had a mastectomy (n = 10), ALND (n = 14), and a history of having a combination of chemotherapy, radiotherapy, and endocrine therapy (n = 11). Table 1 shows the sample characteristics. Feedback was obtained from 12 of 22 (response rate: 55%) invited multidisciplinary experts. Experts came from four countries (Canada, Denmark, Poland, and the United Kingdom) and included eight plastic surgeons, two breast surgeons, a medical oncologist, and a nurse practitioner.

Table 2 provides a summary of scale item revisions during each round. Overall, the scales’ content was deemed easy for participants to understand. Participants only specifically asked for clarification for two items, both of which were dropped. The instructions were generally easy to understand. To the Appearance scale, after Round 3, we added instructions to make sure that women who wear an arm sleeve know to answer thinking of how their arm looks without the arm sleeve.

**Table 2:** Step 2—Pilot Testing 1—summary table showing changes to each scale

Scales	Decisions	Round 1	Round 2	Round 3	Field-test
Symptom		n = 18	n = 19	n = 19	n = 20
	Retain	5	18	19	15
	Revise	10	1	0	0
	Drop	3	0	0	5
	Add	4	0	1	0
Function		n = 7	n = 14	n = 18	n = 19
	Retain	0	0	17	12
	Revise	5	14	1	0
	Drop	2	0	0	7
	Add	9	4	1	0
Appearance		n = 11	n = 14	n = 16	n = 14
	Retain	10	11	13	10
	Revise	1	1	1	0
	Drop	0	2	2	4
	Add	3	4	0	0
Impact		n = 9	n = 11	n = 11	n = 11
	Retain	5	6	11	0
	Revise	0	1	0	0
	Drop	4	4	0	0
	Add	6	4	0	0
Information		n = 12	n = 18	n = 20	n = 13
	Retain	3	17	13	9
	Revise	9	1	0	0
	Drop	0	0	7	4
	Add	6	2	0	0
Psychological		NA	n = 19	n = 19	n = 19
	Retain		19	19	12
	Revise		0	0	0
	Drop		0	0	7
	Add		0	0	0
Arm sleeve		NA	n = 11	n = 15	n = 14
	Retain		8	14	10
	Revise		1	0	0
	Drop		2	1	4
	Add		6	0	0

After Round 1, two new scales were added to measure satisfaction with arm sleeve and psychological function (see Table 2 in Supplementary Material Files, Appendix 4 for patient quotes). Both concepts were identified as important concerns during the first round of cognitive interviews and considered a gap by the research team. Data from the initial qualitative and cognitive interviews were used to create content for the scale. A summary of changes is provided in Table 2. The field-test version included 110 items: symptoms (n = 20), function (n = 19), appearance (n = 14), life impact (n = 11), psychological (n = 19), information (n = 13), and arm sleeve (n = 14). This version of the LYMPH-Q UE was translated into Danish [23] following best practice guidelines [25, 26], and the content validity of the scales was established.

The psychometric findings for the new scales are published elsewhere [14]. Briefly, data were collected from 3222 patients (n = 2858, Denmark; n = 364, U.S.) as part of an international field-test study. One scale (life impact) was dropped due to poor psychometric performance. The final six scales measured symptoms, function, appearance, psychological function, and satisfaction with information and with arm sleeves. Table 4 shows the characteristics of the six LYMPH-Q UE scales, including the number of items, response options, recall period, and Flesch-Kincaid grade reading level.

### Step 3: Concept Elicitation 2—lymphedema worry and impact on work concepts

A total of 12 interviews were completed. The participants were aged between 35 and 72 years. Table 1 shows the demographic and clinical characteristics of this sample. In addition to the concepts of interest (see Table 3 in Supplementary Material Files, Appendix 4 for illustrative patient quotes), participants elaborated on other HRQL issues that mattered to them. The interview data supported the content of the six LYMPH-Q UE scales developed in Step 1 and 2.

### Step 4: Focus Groups—content validity for all HRQL concepts

Four focus group sessions were held with a total of 16 participants (BCRL, n = 14) with UE lymphedema; the number of women who took part in each focus group was six (Session 1), four (Session 2), four (Session 3), and three (Session 4). Two participants also had leg lymphedema (one each in Session 1 and 4). For these participants, information about their leg lymphedema was not coded. For two participants, their UE lymphedema was related to ovarian cancer and melanoma treatment. The focus group sample was aged between 35 and 74 years. Twelve patients had a complete axillary lymph node dissection, two had sentinel lymph node biopsy, and two others were unsure of the type of lymph node surgery they had received. One participant had lymphedema in both arms. All participants wore a compression sleeve/bandage on their arm, and most had manual lymphatic drainage and did exercise prescribed by a physiotherapist or other healthcare professional. Table 1 shows the sample characteristics of the focus group participants. Codes on the impact of lymphedema on HRQL (e.g., physical symptoms, social life, work, appearance, emotional distress, and sexual well-being) were used in the new scale development and to add further evidence of content validity for existing scales (See Tables 1–3 in Supplementary Material Files, Appendix 4 for illustrative patient quotes).

### Step 5: Pilot Testing 2—for new lymphedema worry and impact on work scales

Cognitive debriefing interviews were conducted with seven patients with BCRL from the focus group cohort (January–March 2022) to assess the two new scales’ relevance, comprehensiveness, and comprehensibility. Five interviews were conducted in Round 1, one interview in Round 3, and one interview in Round 4. Twelve clinical experts also reviewed the scales and provided feedback on item relevance and comprehensiveness (Round 2). Table 3 provides a summary showing multiple revisions to the scale instructions, response options, and items in response to the feedback received by patients and experts. A total of 42 items were reviewed in Round 1. Of these, 26 were retained, 12 were revised, four were dropped, and one question was added. In Round 2, three items were dropped, one was added, and all the remaining items were revised to change the verb tense. In Round 3, two additional items were added and one revised, and in Round 4, one item was dropped while the rest were retained. The final field test version of the scales includes 17 items in the impact on work scale and 21 in the worry scale. The response options were modified from agreement to frequency (never, rarely, sometimes, often, always) based on feedback in Round 2. The recall period of “in the past week” was included for the lymphedema worry scale. The wording of the scale instructions was revised accordingly. Table 4 shows a summary of all LYMPH-Q UE scales.

**Table 3:** Step 5—Pilot Testing 2—summary table showing changes to impact on work and lymphedema worry scales

Scales	Decisions	Round 1	Round 2	Round 3	Round 4
Impact on work		n = 17	n = 16	n = 16	n = 17
	Retain	10	0	16	17
	Revise	5	16	0	0
	Drop	2	0	0	0
	Add	1	0	1	0
Lymphedema worry		n = 25	n = 23	n = 21	n = 22
	Retain	16	0	20	21
	Revise	7	20	1	0
	Drop	2	3	0	1
	Add	0	1	1	0

**Table 4:** Description of LYMPH-Q | Upper Extremity Scales

Name of scale	Items	Response options	Recall period	Flesch-Kincaid
**Health-related quality of life**				
Appearance	10	Extremely → not at all bothered	Now	2.4
Function	12	Extremely → not at all difficult	Past week	4.2
Psychological	12	Always → never	Past week	12.0
Symptoms	15	Severe → none	Past week	4.4
Impact on work	17	Never → always	Most recent	4.4
Lymphedema worry	21	Never → always	Past week	4.4
**Experience of care**				
Information	9	Dissatisfied → satisfied	N/A	7.4
Treatment				
Arm sleeve	10	Dissatisfied → satisfied	Most recent	2.2

*N/A* Not Applicable

## Discussion

PROMs are increasingly used in clinical research and practice. When choosing a PROM, high content validity is vital to measuring change following an intervention. The in-depth qualitative interviews with patients with upper extremity lymphedema and the modular approach used to develop the LYMPH-Q UE allowed for a systematic and iterative process of developing and refining scales. It enabled us to generate additional qualitative evidence to demonstrate the content validity of the LYMPH-Q UE scales developed in Steps 1 and 2, consequently ensuring that the scales remain “fit for purpose” in different subsets of patient participants. The modular approach also facilitated flexibility to developing and validating new scales to fill conceptual gaps in measurement as they were identified.

This iterative approach to the development of a PROM and the demonstration of content validity is seldomly documented in the health services research literature, although common in education measurement. Content validity is the most important measurement property of a PROM, as without it, other measurement properties such as reliability, validity, and responsiveness are meaningless. However, evaluation of content validity should not be a one-time process. It is typically examined during PROM development and pilot testing; however, this research and our prior work [27] show that content validity should be periodically reviewed, especially if new treatments become available or clinical knowledge evolves, causing changes in the content domain. Furthermore, as was the case with LYMPH-Q UE, feedback from patients and LYMPH-Q users identified gaps in the measurement of lymphedema worry and impact of lymphedema on work life, leading to the development of two new scales. Hence, routinely assessing the PROM’s alignment with the content domain helps maintain the quality and relevance of measurement.

The readability of the LYMPH-Q UE was assessed using the established Flesch-Kincaid (FK) grade level. The FK grade level indicates the comprehension difficulty of written text and provides a numerical score corresponding to the U.S. school grade level [28]. The FK grade level has been criticized for its focus on sentence length and syllable count, as well as its lack of accounting for the structural and semantic complexity of sentences. Further, similar to other commonly used readability scores, such as the Simple Measure of Gobbledygook (SMOG) readability formula and the Coleman-Liau Index, the FK grade level has been criticized for oversimplifying the complexity of reading comprehension [28]. Nonetheless, the FK grade level is a commonly used measure and can be generated in Microsoft Word (i.e., without complex programs or software). It is recommended that more than one readability score be used to evaluate the reading grade level of written text; however, a comprehensive readability analysis of the LYMPH-Q UE is beyond the scope of this paper.

Our study had some limitations. The initial qualitative sample involved women from only the U.S. and Canada. The cognitive debriefing interviews included Danish women; however, the interviews in Danish were not audio-recorded for pragmatic reasons, as translation is time-consuming and expensive. While the LYMPH-Q UE’s content validity was demonstrated in U.S., Canada, and Denmark, it is recommended that the content validity should be re-evaluated when the LYMPH-Q UE is used in a different context (e.g., country, language) and different population (i.e., non-BCRL) [12, 13]. Another limitation was the lack of any clinical measure of the severity of arm lymphedema for participants in the qualitative interviews, cognitive debriefing interviews, and focus groups. However, our study included women with self-reported mild to severe lymphedema and women for whom BCRLwas managed conservatively or surgically.

## Conclusion

The six scales of LYMPH-Q UE module were field tested and are free for not-for-profit clinical research, clinical care, and quality improvement initiatives through http://www.qportfolio.org. The new LYMPH-Q UE lymphedema worry and impact on work-life scales are currently being field-tested. This study’s innovative and iterative approach to content validation demonstrates that the LYMPH-Q UE is a comprehensive measure that includes important concepts relevant to patients with UE lymphedema.

### Electronic supplementary material

Below is the link to the electronic supplementary material.

Supplementary Material 1

## Data Availability

The datasets used and/or analysed during the current study available from the corresponding author on reasonable request.

## List of abbreviations

BCRL: Breast cancer-related lymphedema
BWH: Brigham and Women’s Hospital
DK: Denmark
HRQL: Health-related quality of life
JCC: Juravinski Cancer Center
LYMQOL: Lymphedema Quality of Life Questionnaire
Lymph-ICF: Lymphedema Functioning, Disability and Health Questionnaire
LSIDS-A: Lymphedema Symptom Intensity and Distress Survey Arm
MSK: Memorial Sloan-Kettering
PROMs: Patient-reported outcomes measures
RMT: Rasch Measurement Theory
TGH: Toronto General Hospital
UE: Upper extremity
ULL-27: The Upper Limb Lymphedema 27 Questionnaire
U.S.: United States

## References

[1] DiSipio T, Rye S, Newman B, Hayes S (2013) Incidence of unilateral arm lymphoedema after breast cancer: a systematic review and meta-analysis. Lancet Oncol 14(6):500–515 23540561 10.1016/S1470-2045(13)70076-7

[2] Armer JM, Hulett JM, Bernas M, Ostby P, Stewart BR, Cormier JN (2013) Best practice guidelines in assessment, risk reduction, management, and surveillance for post-breast cancer lymphedema. Curr Breast Cancer Rep 5(2):134–144 26246870 10.1007/s12609-013-0105-0 PMC4523280

[3] Sierla R, Dylke ES, Kilbreath S (2018) A systematic review of the outcomes used to assess upper body lymphedema. Cancer Invest 36(8):458–473 30289283 10.1080/07357907.2018.1517362

[4] Greene AK, Goss JA (2018) Diagnosis and staging of lymphedema. Semin Plast Surg 32(1):12–16 29636648 10.1055/s-0038-1635117 PMC5891654

[5] Beelen LM, van Dishoeck A-M, Tsangaris E, Coriddi M, Dayan JH, Pusic AL, et al. (2021) Patient-reported outcome measures in lymphedema: a systematic review and COSMIN analysis. Ann Surg Oncol 28(3):1656–1668 33249519 10.1245/s10434-020-09346-0 PMC8693252

[6] Schaverien MV, Offodile AC, Gibbons C (2021) Patient-reported outcome measures in lymphedema: a systematic review and COSMIN analysis. Ann Surg Oncol 28(3):1273–1274 33211230 10.1245/s10434-020-09348-y

[7] Keeley V, Crooks S, Locke J, Veigas D, Riches K, Hilliam R (2010) A quality of life measure for limb lymphoedema (LYMQOL). J Lymphoedema 5(1):26–37

[8] Launois R, Alliot F (2000) Quality of life scale in upper limb lymphoedema–a validation study. Lymphology 33(Suppl):266–274

[9] Devoogdt N, Van Kampen M, Geraerts I, Coremans T, Christiaens M-R (2011) Lymphoedema functioning, disability and health questionnaire (Lymph-ICF): reliability and validity. Phys Ther 91(6):944–957 21493748 10.2522/ptj.20100087

[10] De Vrieze T, Vos L, Gebruers N, De Groef A, Dams L, Van der Gucht E, et al. (2019) Revision of the lymphedema functioning, disability and health questionnaire for upper limb lymphedema (Lymph-ICF-UL): reliability and validity. Lymphat Res Biol 17(3):347–355 30759059 10.1089/lrb.2018.0025

[11] Ridner SH, Dietrich MS (2015) Development and validation of the lymphedema symptom and intensity survey-arm. Support Care Cancer 23(10):3103–3112 25752884 10.1007/s00520-015-2684-y PMC4554806

[12] U.S. Food and Drug Administration. Patient-reported outcome measures: use in medical product development to support labeling claims guidance for industry. 2009 https://www.fda.gov/regulatory-information/search-fda-guidance-documents/patient-reported-outcome-measures-use-medical-product-development-support-labeling-claims. 10.1186/1477-7525-4-79 PMC1629006 17034633

[13] Mokkink LB, Terwee CB, Patrick DL, Alonso J, Stratford PW, Knol DL, et al. (2010) The COSMIN checklist for assessing the methodological quality of studies on measurement properties of health status measurement instruments: an international Delphi study. Qual Life Res 19(4):539–549 20169472 10.1007/s11136-010-9606-8 PMC2852520

[14] Klassen AF, Tsangaris E, Kaur MN, Poulsen L, Beelen LM, Jacobsen AL, et al. (2021) Development and psychometric validation of a patient-reported outcome measure for arm lymphedema: the LYMPH-Q Upper Extremity Module. Ann Surg Oncol 28(9):5166–5182 34224044 10.1245/s10434-021-09887-y PMC8349351

[15] Patrick DL, Burke LB, Gwaltney CJ, Leidy NK, Martin ML, Molsen E, et al. (2011) Content validity—establishing and reporting the evidence in newly developed patient-reported outcomes (PRO) instruments for medical product evaluation: ISPOR PRO good research practices task force report: part 2—assessing respondent understanding. Value Health 14(8):978–988 22152166 10.1016/j.jval.2011.06.013

[16] Patrick DL, Burke LB, Gwaltney CJ, Leidy NK, Martin ML, Molsen E, et al. (2011) Content validity—establishing and reporting the evidence in newly developed patient-reported outcomes (PRO) instruments for medical product evaluation: ISPOR PRO good research practices task force report: part 1—eliciting concepts for a new PRO instrument. Value Health 14(8):967–977 22152165 10.1016/j.jval.2011.06.014

[17] Andrich D (2011) Rating scales and Rasch measurement. Expert Rev Pharmacoecon Outcomes Res 11(5):571–585 21958102 10.1586/erp.11.59

[18] Rasch G (1960) Studies in mathematical psychology: i. Probabilistic models for some intelligence and attainment tests. Nielsen & Lydiche

[19] Thorne S (2016) Interpretive description: qualitative research for applied practice. Routledge

[20] Kaur M, Pusic AL, Cano SJ, Xie F, Bordeleau L, Zhong T, et al. (2020) International phase 1 study protocol to develop a health state classification system for a preference-based measure for women with breast cancer: the BREAST-Q utility module. BMJ Open 10(1):e034451 31915176 10.1136/bmjopen-2019-034451 PMC6955575

[21] Kaur MN, Klassen AF, Xie F, Bordeleau L, Zhong T, Cano SJ, et al. (2021) An international mixed methods study to develop a new preference-based measure for women with breast cancer: the BREAST-Q utility module. BMC Womens Health 21(1):1–17 33407389 10.1186/s12905-020-01125-z PMC7789506

[22] Willis GB (2005) Cognitive interviewing in practice: think-aloud, verbal probing and other techniques. Cognitive interviewing: a tool for improving questionnaire design. Sage Publications, London, pp 42–63

[23] Madsen CB, Poulsen L, Jørgensen MG, Lorenzen MM, Tsangaris E, Klassen A, et al. (2021) Advanced translation and cultural adaption of the LYMPH-Q Upper Extremity Module from English to Danish. Eur J Plast Surg 45(6):1–634728900

[24] Kaur MN, Cornacchi SD, Klassen AF, Haykal S, HIrcock C, Mehrara BJ, et al. (2023) Ensuring patient centeredness in Upper Extremity Lymphedema research: identifying patient-prioritized agenda and preferences. J Plast Reconstr Aesthet Surg 83:326–333 10.1016/j.bjps.2023.04.036 37302238

[25] Organization WH (2009) Process of translation and adaptation of instruments. Geneva: World Health Organization; 2016 (http://www.who.int/substance_abuse/research_tools/translation/en/, accessed 20 Oct 2022)

[26] Wild D, Grove A, Martin M, Eremenco S, McElroy S, Verjee-Lorenz A, et al. (2005) Principles of good practice for the translation and cultural adaptation process for patient-reported outcomes (PRO) measures: report of the ISPOR task force for translation and cultural adaptation. Value Health 8(2):94–104 15804318 10.1111/j.1524-4733.2005.04054.x

[27] Kaur MN, Chan S, Bordeleau L, Zhong T, Tsangaris E, Pusic AL, Cano SJ, Klassen AF (2023) Re-examining content validity of the BREAST-Q more than a decade later to determine relevance and comprehensiveness. J Patient-Rep Outcomes 7(1):1–1 37022647 10.1186/s41687-023-00558-y PMC10079800

[28] Benjamin RG (2012) Reconstructing readability: recent developments and recommendations in the analysis of text difficulty. Educ Psychol Rev 24:63–88 10.1007/s10648-011-9181-8

