# Supplementary material for: Iterative qualitative approach to establishing content validation of a patient-reported outcome measure for arm lymphedema: the LYMPH-Q Upper Extremity Module
Source: J Patient Rep Outcomes. 2024 Jun 26;8:63. doi: 10.1186/s41687-024-00701-3 (PMC11208340; doi:10.1186/s41687-024-00701-3)
Supplement: Supplementary file 1 — Supplementary Material 1 [file 41687_2024_701_MOESM1_ESM.docx]

**Supplementary Material Files - Appendix 1: Semi-structured interview guide – breast cancer**

| Today’s date / Interview location / Interviewer / Participant ID number  *start recording*  Pre-interview script: Hello <insert participant name>, I want to thank you for taking the time to speak with me today. I would like to talk to you about your breast cancer journey and how breast cancer and the treatments that you have received to-date impacted your quality of life. I am particularly interested in hearing about which aspects of your health and quality of life that have been the most and least important to you during your cancer experience. Do you have any questions before we begin? <allow the participant to ask any additional questions regarding the study or the study procedure> Do you consent to participate in the study? <Once participant consents proceed with the interview>.  Interview Questions   1. Can you tell me about the events leading up to and including your breast cancer diagnosis? 2. Can you tell me what being a breast cancer survivor/breast cancer patient has been like for you? 3. What kinds of treatments have you had/will you have in the future? 4. Describe any symptoms or adverse effects you experienced from breast cancer and/or its treatment (e.g., pain, complications, etc.)? Out of the symptoms you mentioned, can you tell me which have had the biggest/smallest impact on quality of life, and why? 5. How did breast cancer and/or its treatment change your appearance (in clothes, naked)? Of the changes in your appearance, which are the most/least important and why? 6. Describe any physical concerns caused by breast cancer and/or its treatment. Out of the problems you mentioned, can you tell me which are the most/least important and why? 7. Describe any activity limitations caused by breast cancer and/or its treatment(s). Out of the activity limitations you mentioned, can you tell me which are the most/least important and why? 8. Describe any emotional concerns caused by breast cancer or as a result of breast cancer treatment(s) Out of the problems you mentioned, can you tell me which are the most/least important and why? 9. Describe any concerns with sexual life caused by breast cancer or as a result of breast cancer treatment(s). Out of the sexual concerns you mentioned, can you tell me which are the most/least important and why? 10. Describe any problems with your social or work-related relationships and roles caused by breast cancer or as a result of breast cancer treatment(s). Out of the social concerns you mentioned, can you tell me which are the most/least important and why? 11. Can you describe any other concerns or issues you experienced that we have not already covered? (e.g., spiritual, cancer worry, etc.) 12. Thinking back over what you have talked about in this interview, what would you say are the most/least important aspects of your quality of life impacted by breast cancer and/or its treatment.   *stop recording* |
| --- |

**Supplementary Material Files - Appendix 2: Semi-structured interview guide – breast cancer related arm lymphedema**

| Today’s date / Interview location / Interviewer / Participant ID number  *start recording*  Pre-interview script: Hello <insert participant name>, I want to thank you for taking the time to speak with me today. I would like to talk to you to learn about how your lymphedema and is treatments has had on your quality of life. Do you have any questions before we begin? <Allow the participant to ask any additional questions regarding the study or the study procedure> Do you consent to participate in the study? <Once participant consents proceed with the interview>.  Interview Questions   1. What kinds of treatments for breast cancer have you had/will you have in the future? 2. Can you tell me about your lymphedema, when it started, the first thing you noticed, how it has progressed, and what the outlook is like? 3. Can you tell me about any treatments you have had for your lymphedema (e.g., medication, surgery)? 4. Please describe in detail any physical symptoms/sensations you experienced with the lymphedema (e.g., pain, discomfort, swelling, heaviness, temperature changes, etc.)? Can you tell me which are the most/least important and why? Probe: location of symptoms – hand/fingers/shoulder. Do they vary for scenarios (time of day, weather, etc.)? What does it feel like (intensity, frequency, severity etc.)? 5. What impact/interference does it have on activities (self-care, ADL, sleep, etc.)? Probe all possible arm and hand activities that are affected. Can you tell me which are the most/least important and why? Probe for accommodation and adaption strategies. 6. Can you describe any activities you avoid to take care for your lymphedema so it doesn’t get worse? Are there activities you should avoid but don’t? Probe for protection against injury, insect bites, road or air travel, etc. 7. Does lymphedema affect your body image? Probe for feelings of self-conscious, embarrassed, concealment behavior, impact on clothing choice, changes in the nails and skin (color, texture, etc.) 8. Describe any emotional concerns caused by lymphedema. Out of the problems you mentioned, can you tell me which are the most/least important and why? Probe for coping strategies 9. Describe any concerns with sexual life caused by lymphedema. Out of the sexual concerns you mentioned, can you tell me which are the most/least important and why? 10. Describe any problems with your social or work-related relationships and roles caused by lymphedema. Out of the social concerns you mentioned, can you tell me which are the most/least important and why? Probe for accommodations at work (assistance, modified duties, etc.) and home (assistance, etc.) 11. Describe any financial impact of having lymphedema. Probe: insurance and health care cost issues 12. Can you tell me about information and advice you have received from healthcare providers about your lymphedema and how to look after it (what you can and cannot do, etc.)? What kind of information do other people with lymphedema need to know? 13. Can you describe any other concerns or issues you experienced that we have not already covered? 14. Thinking back over what you have talked about in this interview, what would you say are the most/least important aspects of your quality of life impacted by lymphedema.   Post Interview script: Thank you for taking time to meet with me today to share your views, experiences and stories. Do you have any final questions before we part?  *stop recording* |
| --- |

**Supplementary Material Files - Appendix 3: Focus group interview guide – breast cancer related arm lymphedema**

| Today’s date / Interview location / Interviewer / Participant ID number  *start recording*  Pre-session script: Hello all, I want to thank you for taking the time to speak with me today. As you are aware, our research team is working towards developing a patient-reported instrument for women diagnosed with breast cancer-related lymphedema for use in clinical practice and research. At the session today, we would like to understand what quality of life outcomes matter the most to you and get your feedback on what a patient-led study design to study lymphedema would look like.  Do you have any questions before we begin? <allow the participants to ask any additional questions regarding the study or the study procedure> Do you consent to participate in the study? <once participants consent, proceed with the interview>  Focus group questions  **Impact on HRQOL**   - How has lymphedema affected your quality of life? (Probe: physical symptoms, daily activities, social life, work, appearance, emotional distress, sexual well-being)   **Factors influencing decision-making**   - What were your reasons for seeking/not seeking lymphedema surgery? (Probe: burden of conservative treatment, caregiver impact, financial impact)   **Understanding outcomes that matter**   - What are the most important outcomes that you were hoping to achieve from surgery? (Probe: recurring infections, anxiety, symptoms, function (daily vs. leisure, social, work, travel), appearance (clothes fit, body image)   **Sustained patient engagement**  Some of you may have completed surveys that are specially designed to measure outcomes that matter to you during your care at the cancer center or for your lymphedema care. Typically, clinicians and researchers collect these data at regular time-points to assess the impact of the disease or the treatment.   - What do you think are some of the challenges in completing surveys on a regular basis? (Probe: format, burden, reasonable time-points)   **Patient-led study design**   - If there was a study of surgery to treat lymphedema, would you be willing to take part in that study? How would you feel if access to the surgery was randomized? By randomized, we mean that a computer program would be used to decide if a participant will get the surgery for lymphedema or continue with current management? (Probe: why, why not)   Post Interview script: Thank you for taking time to meet with me today to share your views, experiences and stories. Do you have any final questions before we part?  *stop recording |
| --- |

**Supplementary Material Files - Appendix 4**

**Table 1. Patient-reported experiences of physical symptoms, physical function, appearance, satisfaction with information related to upper extremity lymphedema (LE).**

| **Domain** | **Theme** | **Sample quotes** |
| --- | --- | --- |
| **Physical Symptoms** | Pain | “…I would feel pain if I moved my arm a lot,” Step 1 - Q3*  “…it throbs.” Step 1 - Q11  “…it is like often quite painful.” Step 3 - Q22  “…I also had pain in my lower arm.” Step 4 - FG1  “I was in excruciating pain all the time…” Step 4 - FG4  “The swelling hurts.” Step 4 - FG4 |
|  | Stiff | “I notice it gets a bit stiff like when I go to bend my arm.” Step 2 – Cog5  “…every morning I have to move my hands to get them moving, like they are very stiff” Step 3 – Q16  “So I just think it felt stiff” Step 4 - FG4 |
|  | Symptoms disturb sleep | “Sleeping at night, sometimes it bothers me.” Step 1 - Q2  “Any time I move [while sleeping], I wake up. It’s just very uncomfortable, very painful.” Step 1 - Q4  “I couldn’t sleep on my left side.” Step 1 - Q9  “Sleeping at night, sometimes it bothers me” Step 2 – Cog2  “I had trouble sleeping actually when it was bad” Step 3 - Q23 |
|  | Aching | “It’s just sometimes my arm will start aching” Step 1 - Q11  “The aching is I would say often.” Step 2 - Cog1  “All day long. Every day. It’s like burning….achy pain.” Step 4 - FG2  “I describe it as having a toothache in the arm.” Step 4 - FG3 |
|  | Numb/tingling pins and needles | “I get that pins and needles, numbness feeling in my right hand.” Step 3 - Q17  “…my left hand was just numb and swollen.” Step 3 - Q23  “It was mostly if my arm was bad, the tingling was really uncomfortable…” Step 3 - Q23  “…I have a lot of numbness in my fingers” Step 4 - FG2  “…it’s very sort of burning and tingling and numb.” Step 4 - FG2 |
|  | Clumsiness | “…so I would drop things and I would not be able to grasp things properly.” Step 3 - Q22  “…my fingers were clumsy.” Step 3 - Q23  “…my left hand wouldn’t cooperate for fine motor tasks.” Step 3 - Q23  “I’m always afraid that it’s [glass] going to slip out of my grip.” Step 4 – FG1  “…I feel like I’m going to drop things.” Step 4 - FG1 |
|  | Tired | “The worst thing about my lymphedema is my arm swelling and the fatigue in that arm.” Step 1 - Q11  “The [arm] tightness and the fatigue affect me the most.” Step 1 - Q14  “Sometimes that feels, you know, a little more fatigued” Step 2 - Cog1  “…it [arm] just tires very easily” Step 4 - FG1 |
|  | Weak | “It is still much weaker.” Step 1 - Q9  “And weakness in my arm I would say often. It’s not as strong as my other arm.” Step 2 - Cog1  “I am still weaker in that arm and it does drive me a little crazy…” Step 3 - Q27  “…you can definitely feel the weakness on the left side.” Step 4 - FG4 |
|  | Heavy | “It feels heavy.” Step 1 - Q1  “It feels full. It feels heavier.” Step 1 - Q4  “…it just felt….as I say, a dead weight. It was heavy.” Step 1 - Q19  “The arm is just too heavy. I can’t keep it up.” Step 4 - FG1  “There was a heaviness and a hardness about it that made it more painful.” Step 4 - FG3 |
|  | Swelling | “And the swelling was in my hand and mostly my lower arm.” Step 1 - Q13  “…if you use it excessively, then you will have more swelling.” Step 2 – Cog1  “It’s swollen” Step 2 – Cog5  “…the swelling was getting more and more.” Step 3 - Q16  “And because it’s fluid, it moves. Sometimes my swelling is worse here.” Step 4 - FG2 |
| **Physical Function** | Putting on/off clothes | “I would say particularly my bra [was difficult to put on]. And then for a while it was my shirts.” Step 2 - Cog 4  “Dressing was difficult…difficulty doing up buttons.” Step 3 - Q23  “[Participant]: I would say particularly my bra. And then for a while it was my shirts. [Interviewer]: Lifting your arm up to get the shirt on? [Participant]: Yup.” Step 1 - Q4 |
|  | Washing hair | “…getting a brush through my hair. It was hard sometimes.” Step 1 - Q3  “That was hard [washing hair].” Step 2 - Cog4  “I have long curly hair so when I’m in the shower and you have to wash your hair, you can definitely feel the weakness on the left side.” Step 4 - FG4 |
|  | Reaching across yourself | “This was very tender and pulling that seatbelt on…” Step 1 – Q4  “Like if I’m in the driver’s seat and I’m reaching around to get something. Or if…I have my arm around someone in a photo…that’s actually the hardest.” Step 2 - Cog4  “Even things like putting on your seatbelt…that hurts. Can’t do that.” Step 4 - FG4 |
|  | Gripping/holding | “I just don’t have the grip and the dexterity.” Step 1 - Q4  “[Interviewer] So you avoid grabbing…do you find your grasp is weaker as well? [Participant]: Yes.” Step 2 - Cog1  “Being able to open jars myself, like if something was really tough, because I can’t twist it” Step 3 - Q20  “I would not be able to grasp things properly.” Step 3 - Q22  “No, I just don’t have as much feeling. It’s just there is no…the grip is not there.” Step 4 - FG1 |
|  | Using hand/fingers | “Doing puzzles and stuff like that is very difficult for me to do now.” Step 3 - Q22  “But even sitting to do crafts and stuff, my left hand wouldn’t cooperate for fine motor tasks.” Step 3 - Q23  “And I used to be able to type 200 words a minute. And of course, now I cannot.” Step 3 - Q23  “But there is a manual dexterity problem.” Step 4 - FG2 |
|  | Reaching overhead | “Yes, there is difficulty reaching overhead.” Step 2 - Cog1  “I can’t stretch my arm all the way up to grab something off the top shelf.” Step 3 - Q20  “Well, I can’t raise my arm over my head anymore.” Step 4 - FG4 |
|  | Holding/lifting | “…you just make sure your grocery bags aren’t too full. You make sure that you’re not really carrying the heavy stuff.” Step 1 - Q2  “There is difficulty lifting heavy objects.” Step 2 – Cog1  “There are certain things, like carrying things up and down stairs…it can’t be too heavy.” Step 3 - Q19  “I couldn’t carry a laundry basket.” Step 3 – Q23  “I can’t lift things with my left hand on its own.” Step 4 - FG4 |
|  | Doing household chores | “I could sweep the floor for a little while, but then…my right arm is tired.” Step 1 - Q9  “I just remember being in my kitchen and being really frustrated to not be able to do a lot of things.” Step 2 - Cog3  “Or making the bed, that’s hard is if the blankets are heavy.” Step 2 - Cog4  “Cooking was difficult.” Step 3 - Q23 |
|  | Physical activities – moving arm | “I used to love to bowl, and I have not bowled since all this.” Step 1 - Q11  “I avoid lifting things, gardening, that kind of thing where I’m using my arm too much and it’s going to affect the swelling of it.” Step 3 - Q16  “Well, I don’t golf anymore.” Step 3 – Q17  “I ended up canceling my gym membership, because I used to do a lot of weight-lifting and body pump classes which do the liftings over your head which I cannot do anymore.” Step 4 - FG4 |
| **Appearance** | People seeing arm | “…then people look at my arm and that just drives me crazy. I just don’t want to be noticed.” Step 1 - Q11  “Everyone stares. Everyone asks what happened to me. And I can't keep answering the question. I just back away.” Step 1 - Q14  “I am self-conscious. People see the sleeve and they ask.” Step 1 - Q15  “So, when you’re meeting somebody and they are staring at your hand or your arm because it looks a little strange. Or you have a glove or a wrap on it or whatever it is…it’s not pleasant to deal with. It makes me not want to go meet people.” Step 3 - Q22  “I think we all get tired of people making comments about how our limbs are puffy.” Step 4 - FG2 |
|  | Hiding arm | “[Interviewer]: Why do you wear full sleeves when you are going out for dinner? [Participant]: Well just to hide the sleeve on my arm. Because it’s not attractive.” Step 1 - Q6  “…if I’m out in public…I will wear like a long sleeve or a jacket or just something that it’s just not that conspicuous.” Step 1 - Q11  “Yeah, I would say that it’s a daily consciousness in terms of dressing in a way to cover your arm. I do. I always do.” Step 2 – Cog2  “[Interviewer]: And you try to conceal it [arm] with clothing? [Participant]: With clothing, yes.” Step 4 - FG4 |
|  | Photos | “I usually try to hide my right arm in the photograph…It makes me look fat, which I’m not.” Step 1 - Q10  “I don’t like selfies. I don’t like doing individual pictures…” Step 1 - Q11  “When we are doing a family photograph, I always stand and hide my arm.” Step 4 - FG3 |
|  | Swelling noticeable | “…this hand is so much more puffy than this one.” Step 1 - Q4  “…he [spouse] was looking at me from the back, he goes, your arm is bigger than the other.” Step 1 - Q6  “But people notice my hand [swelling] more than my arm.” Step 2 - Cog2  “You can definitely see that it is a little bit…like swollen looking and it’s puffed out.” Step 4 - FG4 |
|  | Arm size | “It’s a big swollen arm.” Step 1 - Q11  “My arm at its worst is 62% bigger. 62% bigger is pretty bad.” Step 4 - FG2  “It’s almost like my hand is this gigantic lobster claw.” Step 4 - FG2  “My arm is 3 sizes too big” Step 4 - FG4 |
|  | Clothing | “But sometimes you get longer sleeves that don’t fit because your arm is bigger.” Step 1 - Q3  “…My left arm is tight in the sleeve or if the sleeve is baggy then people say what’s wrong with your arm?” Step 3 - Q16  “Well, it’s just hard to find things that fit because your arm is swollen…I always have to go one size up, so it’s big everywhere else where it doesn’t have to be.” Step 3 - Q24  “It [shirt] has to be bigger and baggier to accommodate for the swelling in my arm.” Step 3 - Q24  “But I look like this blob all the time, because I’m wearing these fat lazy slob clothes that have no shape, but I can’t have anything that puts any pressure on…like any sort of elastic or anything that sort of has a hemline or down here.” Step 4 - FG2 |
| **Information** | Possibility of LE | “I was always informed that it’s a possibility that lymphedema could happen.” Step 3 - Q19  “And it’s something that…they really don’t tell you anything about.” Step 3 - Q22  “I had been told by my surgeon that there is a possibility that I could get lymphedema, but not a ton more information.” Step 4 - FG4  “And I don’t think to this day that anybody really explained to me what lymphedema really was.” Step 4 - FG4 |
|  | Caring for LE | “They were the ones who really taught me how to wrap my arm and give me the exercises and teach me how to massage myself. And gave me the tools that I needed to take care of myself.” Step 1 – Q15  “No one told me what to do on a daily basis. Like the sleeve and the lotion and the exercises.” Step 2 – Cog1  “And she [physiotherapist] gave me tips on what to do and how to stretch out my arm and things like that.” Step 3 - Q19  “I would say that even more anxiety exists because it’s not addressed by the medical team, that you don’t feel confident that what you are doing is the right thing.” Step 3 - Q25  “…my massage therapist did tell me to try to keep your arm elevated at chest level to help with the flow.” Step 4 - FG1 |
|  | Avoid infection | “You are supposed to avoid getting cuts or bitten on that arm, because of the risk.” Step 3 - Q16  “They talked about…I have to be careful not to cut myself or get a burn or just anything with my arm and hand. I have to be very careful because it can become infected quite easily.” Step 3 - Q19  “I had been warned to be careful about things like when I’m chopping stuff, be careful not to get cuts.” Step 3 - Q23 |
|  | LE treatment | “I wish at the outset that I was better aware of what might be needed [for treatment].” Step 2 – Cog2  “And then there are all different types of massage too. And I mean, I don’t know which one is best.” Step 3 - Q16  “I think that it would be great if it was not so much up to women to find their own lymphedema support post-surgery.” Step 3 - Q19  “…not everything was discussed, and it was kind of frustrating, because I found I had to do a lot of self-navigating.” Step 3 - Q23  “I think there is a lack of information for people who could get lymphedema or who have lymphedema. Like what is going on and how to treat it.” Step 4 - FG2 |
|  | Who will monitor LE | “I used to be able to go to the cancer clinic to connect with a lymphedema nurse. And that was really helpful.” Step 1 - Q9  “…There is no lymphedema specialist or doctor that you can go and talk to about it, you know? You just see these massage therapists and that’s it.” Step 3 - Q16  “[Massage therapist] Then talked to me about different exercises to do, how to watch out for different things…” Step 3 - Q19  “She [physiotherapist] has been sort of treating me and she measures my arm.” Step 3 - Q25  “And my doctor did brush it off as well, as no big deal. So it was very frustrating.” Step 3 - Q23 |
|  | Impact of LE on life | “I didn’t realize I was supposed to be wearing my sleeve all the time and the massage therapist said you should, you need it the rest of your life.” Step 1 - Q6  “And I’m sure a lot of new breast cancer patients are really not aware of the extent lymphedema can create, that the risks with it, if you don’t manage it.” Step 3 - Q16  “Like they don’t….you don’t know that this is how it’s going to be forever.” Step 3 - Q22  “Like I had read some things, but I didn’t know that there would be this, not to the extent that it is.” Step 3 - Q22 |

*Denotes the step (1 – 5), type of interview (qualitative, cognitive, focus group) and patient study ID# or Focus group#; Step 1 – Concept Elicitation 1;

Step 2 – Pilot Testing 1; Step 3 – Concept Elicitation 2; Step 4 – Focus Groups; Step 5 – Pilot testing 2; Q – qualitative interview; Cog – cognitive interview;

FG – focus group

**Table 2. Patient-reported experiences of wearing compression sleeve and psychological impact of upper extremity lymphedema.**

| **Domain** | **Theme** | **Sample quotes** |
| --- | --- | --- |
| **Arm sleeve** | Fit/comfort | “Well, it’s not always that comfortable. It’s hot in the summer.” Step 1 - Q11  “Well... it's uncomfortable. I mean, it's tight.” Step 1 - Q13  “There are other times where I find it very frustrating, and I just have to rip off that sleeve because it’s just too much” Step 2 – Cog1  “I couldn’t wear the sleeves for the length of time that they were asking, because it was just so uncomfortable.” Step 4 - FG3  “…the elastic that is around the top of the sleeve, it kind of pinches sometimes and I feel like I’m often adjusting it” Step 4 - FG4 |
|  | Reduce swelling | “My thumb is definitely swollen. I put the glove on for a little bit and it feels better after that.” Step 1 - Q12  “I guess I'm not feeling that it's benefitting me to be worth wearing it.” Step 1 - Q13  “I always have it on...it is therapeutic.” Step 3 - Q16  “That [night sleeve] really helps because it softens the tissue. My arm is very hard…So I find that helps even a lot at nighttime to be wearing that.” Step 4 - FG4 |
|  | Appearance | “It’s not particularly attractive.” Step 1 - Q10  “…and it dirties pretty quickly just from touching papers and things like that. So if anything, I'm a little conscious of how it looks as far as the cleanliness more than anything else.” Step 1 - Q13  “I do wear a tight sleeve every night, one of those thicker ones, looks like a big oven mitt…” Step 1 - Q13  “I don’t want to wear this ugly sleeve.” Step 2 – Cog1  “Because the nude sleeve and glove, it looks strange.” Step 4 - FG4 |
|  | Ease to put on | “It's very difficult [to put on]. And I have more than once punched myself in the face by accident because I was pulling the sleeve up and my hand slipped off what I was pulling and just hit me in the jaw. It's embarrassing.” Step 1 - Q13  “Sometimes I have to get my husband to pull it up high enough. Trying to get the fit right is very difficult.” Step 3 - Q16  “It was difficult to put on.” Step 3 - Q17  “The sleeve could be awkward [to put on].” Step 3 - Q23 |
|  | Impact on physical function | “The sleeve and the gauntlet also make typing more difficult” Step 1 - Q13  “When I have my arm wrapped, I absolutely can't do anything. I can't cook. I can't go to the gym.” Step 1 - Q13  “Also having to wear gloves, opening doors, you know. I can't open doors with it. It doesn't move, because my wrist doesn't bend.” Step 1 - Q14  “It would irritate, because I’m lifting. I had gloves on top and I’m lifting.” Step 3 – Q18 |
|  | Wear sleeve each day | “… the massage therapist said you need it the rest of your life. Well, I don’t like that, but if I have to wear it, I have to wear it.” Step 1 - Q6  “But I am really diligent about wearing my arm sleeve daily and I even wear a night sleeve now.”  Step 2 - Cog1  “So that’s not fun at all. So that’s a real inconvenience. And everywhere you go, you have to take this with you.” Step 3 – Q16  “Having to wear the sleeve is a drain.” Step 3 - Q26 |
|  | Body image | “[Interviewer]: And why do you wear full sleeves when you are going out for dinner? [Participant]: Well just to hide the sleeve on my arm. Because it’s not attractive.” Step 1 - Q6  “I am self-conscious. People see the sleeve and they ask." Step 1 - Q15  “…it makes you self-conscious that you’ve got this going on” Step 2 – Cog1  “At first when I had to wear the glove, I almost felt ashamed…” Step 4 - FG4  “People’s impression if they don’t know you is completely different, like you have a disability or something.” Step 4 - FG4 |
|  | Clothing | “So actually it did really limit what I wore when I had to wear the sleeve all the time.” Step 1 - Q13  “because some long-sleeved things are tight on my arm, especially when you have to have the [compression] sleeve on as well.” Step 2 - Cog1  “I have this great big thing [compression sleeve]…But you can’t go out wearing that. It doesn’t fit under any coat.” Step 4 - FG2  “I just learned to dress differently. Buy clothes that have the longer sleeve [to hide compression sleeve].” Step 4 - FG4 |
| **Psychological** | Anxious/worried | “So, I think I worry about it enough that it affects my life and what I choose to do with my time.” Step 1 - Q13  “I just feel anxiety.” Step 3 - Q25  “So yes, it very much worries me when my forearm is starting to swell. I’m worried about cellulitis.” Step 3 - Q16 |
|  | Afraid | “I think I had…in the beginning a fear of like going to bed at night and waking up and my arm being like 3 times the size.” Step 1 - Q15  “I’m terrified that it is going to get big.” Step 2 - Cog1  “…I think your fear is accelerated because of lymphedema.” Step 3 - Q16  “To tell you the truth, I don’t even garden, because I’m afraid of getting a cut or a scratch.” Step 3 - Q24  “But he [doctor] didn’t address my fears and concerns over it [lymphedema].” Step 4 - FG3 |
|  | Unattractive | “And I’m like, hi I’m a troll. I’m like this Shrek. It’s a horrible thing.” Step 4 - FG2  “But I look like this blob all the time, because I’m wearing these fat lazy slob clothes that have no shape.” Step 4 - FG2  “…I don’t feel attractive at all.” Step 4 - FG3  “I think it’s just a general feeling of not being attractive.” Step 4 - FG3 |
|  | Stressed | “…it was very stressful in the beginning to have this condition.” Step 3 - Q24  “It [lymphedema] causes me more distress than the surgery or the chemotherapy or anything every day. I think it has a huge impact on women’s life.” Step 4 - FG1  “…I find it [lymphedema] really stressful.” Step 4 - FG2  “Just that it’s like all consuming stressful. It causes you a lot of stress” Step 5 - Cog3 |
|  | Depressed | “And then when it flares, you try to keep on a happy face, but in the back of your mind it’s like oh my God, is this ever going to get better or is this now the new normal? That’s really ugly. That’s very depressing…” Step 1 - Q13  “When I first got it, I was so mad and so upset and I was so depressed with having it.” Step 3 - Q21  “Sometimes I don’t even want to think about it, because if I start to think about it then I will be very depressed.” Step 4 - FG2  “[Interviewer]: Does it make you depressed or sad? [Participant]: It does.” Step 4 - FG4 |
|  | Frustrated | “I would say definitely frustrating, because I know I can do more than what I’m doing…now my arm is saying no, you can’t do that no more.” Step 1 - Q11  “I would say yeah, because not being able to take care of the house stuff frustrated me.” Step 2 – Cog3  “It was also very, very frustrating to go from having full use of both hands to having this hand constrained a bit and how it can actively work.” Step 4 - FG1  “So yes frustrated. Demoralized, yes. Covering up, compensating.” Step 4 - FG3  “but when you have huge body changes that impact your activities and daily living, that’s where I find it actually quite annoying and frustrating.” Step 4 - FG4 |
|  | Angry | “But this is why I had a lot of anger. Like really hard core anger over it [getting lymphedema]. Because I followed all of the instructions.” Step 4 - FG2  “Yes, I feel angry all the time.” Step 4 - FG3  “I did get angry this morning because my glove wouldn’t go on well and I just ripped it off and thought ‘this damn thing’.” Step 4 - FG3 |
|  | Irritated | “Definitely it’s annoying in the summer, because there are times when you want to get something done and it’s too hot and humid and I know it will swell up if I go out.” Step 2 - Cog3  “Well it’s a pain in the butt, because you have to….it just puts a….every time you have to do something, you have to think about can I participate in that?” Step 3 - Q24  “But gravity is my enemy...I have a dog and I can’t walk around like this [with arm help up]. So, by the time I get home, I’m feeling it [swelling] again and I’m annoyed.” Step 4 - FG2 |

*Denotes the step (1 – 5), type of interview (qualitative, cognitive, focus group) and patient study ID# or Focus group#; Step 1 – Concept Elicitation 1;

Step 2 – Pilot Testing 1; Step 3 – Concept Elicitation 2; Step 4 – Focus Groups; Step 5 – Pilot testing 2; Q – qualitative interview; Cog – cognitive interview;

FG – focus group

**Table 3. Patient-reported experiences of the impact of upper extremity lymphedema on work and experience of lymphedema worry.**

| **Domain** | **Theme** | **Sample quotes** |
| --- | --- | --- |
| **Impact on Work** | Self-conscious | “At work, I’m supposed to wear a [compression] sleeve all the time, people look at my arm and that just drives me crazy. I just don’t want to be noticed.” Step 1 - Q11  “So sometimes the stigma that people will look and wonder what the heck is wrong with her arm?” Step 3 - Q19  “I was very self-conscious especially at work.” Step 3 - Q23  “And people always asking you, what happened to your arm. I just felt like it was almost an invasion of privacy when you felt that you had to answer that question.” Step 3 - Q24  “I know that the job that I do, I work with a lot of executives and I didn’t want them feeling sorry for me. ‘Oh you poor thing’.” Step 4 - FG1  “Well your arm is large. It’s obviously larger than the other arm. I also have a very swollen hand. I was always aware of it and I could see people looking at it. And sometimes people would ask me what was wrong with my arm. It makes you self-conscious.” Step 5 – Cog6 |
|  | Needed help | “I get help from like lifting heavy things.” Step 1 - Q11  “They [co-workers] would get after me if I was moving something, saying stop that, you shouldn’t be moving that. So, they were aware of it and helped me out whenever they could.” Step 3 - Q19  “…there are certain things that I cannot do, that I did before but I’ve realized that it is a detriment to my arm. So I will not do them. And so I ask my co-worker, like, can you help me lift the paper?” Step 5 - Cog5 |
|  | Needed breaks | “I have to take more breaks.” Step 2 - Cog1  “When I’ve been typing for a long time or sitting in a meeting for a long time. I need to get up and move.” Step 3 - Q27  “I do need to get up every 40 minutes or so [take break]. Certainly, every hour and just shake [arm] or do something” Step 4 - FG2  “If I’m writing a document, the typing always exacerbates the arm, so I would have to take a rest.” Step 5 - Cog6 |
|  | Unable to do work | “Just passing my job off to someone else. And I knew I had to, because I couldn’t.” Step 2 - Cog1  “If I had to go back to my pre cancer job, I honestly don’t know how I could do it, because it was high pressure, fast-paced and I did so much in my job.” Step 3 - Q16  “I was teaching harp. I can’t do harp anymore at all.” Step 3 - Q22  “[Interviewer]: …was lymphedema a factor in that decision to stop working? [Participant]: Absolutely for me the main factor.” Step 4 - FG3 |
|  | Trouble performing work | “And I used to be able to type 200 words a minute. And of course, now I cannot. I never did regain that.” Step 3 - Q23  “And it [wearing compression sleeve] slows down my typing, increases the number of errors that I make.” Step 4 - FG1  “[Interviewer]: Can you describe some of the work-related activities that were difficult to perform? [Participant]: So sitting for long periods of time. Computer work. Writing.” Step 4 - FG3  “So in my case with my typing, even on a good day, I don’t have the amount of flexibility and dexterity in my left hand. So it interferes. I have trouble.” Step 5 – Cog7 |
|  | Reduced work | “I had a restricted amount of work that I was doing.” Step 1 - Q11  “I do way, way less playing and demonstrating for students [music teacher].” Step 3 - Q22  “I have reduced the amount of clients I see in a day...As a result of my lymphedema.” Step 5 – Cog4  “Well if I was writing a large paper or document, I might instead of doing it all in one day, I might spread it out to 2 days because I knew that my arm would become not able to function.” Step 5 – Cog6 |
|  | Worry - lose job | “I was always very conscious of were they going to se me as a liability, because now I wasn’t performing at the same level that I had before all this happened.” Step 3 - Q23 |
|  | Symptoms | “At the beginning of the [work] day it’s fine. The more I do with it, the more tired it gets and the more swelling it starts.” Step 1 - Q11  “Sitting at a desk all day is hard on my arm.” Step 2 - Cog1  “I’m sure it swells more because I am at a computer and working with my arms at that position for so long.” Step 3 - Q16  “…the minute I start typing, it starts to be painful.” Step 4 - FG3  “I do find my arm is really, really stiff by the end of the [work] day and then it is painful.” Step 5 - Cog2 |
|  | Accommodations | “Now I have made a lot of accommodations. I’ve set up an office at home so sometimes I can get up and work from home for a bit before an appointment.” Step 2 – Cog1  “I went back to work with a computer that I purchased that had touch screen and voice recognition to compensate for what I couldn’t do”. Step 3 - Q23  “That’s the advantage of having a flexible work arrangement, so I can kind of fit seeing [massage therapist] into my workday.” Step 3 - Q26  “I’ve made sure that I have the ergonomic keyboard to lessen strain on my arm.” Step 3 - Q27  “So what I do now is if I have to write something, I will dictate it” Step 4 - FG3 |
| **Lymphedema Worry** | Disease progression | “I’m terrified that it [arm] is going to get big” Step 2 - Cog1  “Being frightened and terrified. Imagining how puffy or….I just didn’t have any idea of how big my arm could get and what degree of disability it would offer.” Step 3 - Q17  “I worry that that will get worse, and I won’t be able to use my hands.” Step 3 - Q22  “Yes, I do worry that it will get worse, because when I turn 60 and 70, then hopefully I’m around. That is a worry for sure.” Step 5 - Cog5 |
|  | Infection or injury | “Just getting cut on that hand or that arm is more dangerous. So yeah, you do avoid things that can cause you to get an infection”. Step 3 - Q16  “I do worry about getting an infection. I think it’s always there in the back of my mind.” Step 3 - Q19  “To tell you the truth, I don’t even garden, because I’m afraid of getting a cut or a scratch.” Step 3 - Q24  “I just have to be really careful about getting any sort of cut or abrasion in that arm. It makes me nervous, because I have a very rambunctious dog…so there is worry, constantly.” Step 4 - FG2  “That [injuring arm] is always a worry….Yes. I try and be careful.” Step 5 - Cog3 |
|  | Outdoors (e.g., sunburn, insect bites) | “I have a smaller garden than I normally do because they told me to be careful of [insect] bites.” Step 1 - Q15  “If there are mosquitos outside, I go inside right away, because I don’t want to get bitten.” Step 3 - Q16  “I have sunscreen everywhere, in the house, in the garage, in the backyard. And I’m covered…I try to ensure that I don’t stay out in the sun very long.” Step 3 - Q18  “I had to definitely be careful about making sure that I had sunscreen on and reapplying it, because if I got a sunburn, that would make the lymphedema worse.” Step 3 - Q23  “Just if I’m somewhere and I notice there are mosquitos buzzing around, I get really anxious, wanting to prevent mosquito bites on that arm.” Step 5 - Cog7 |
|  | Impact on activities (eg. exercise, chores) | “I try to avoid doing things with my left arm, simply because that is a trigger for swelling.” Step 1 - Q13  “So I avoid lifting things, gardening, that kind of thing where I’m using my arm too much and it’s going to affect the swelling of it.” Step 2 - Q16  “You have to be careful. Like if you are working out, you don’t want to push it too far because you can feel the difference in that arm.” Step 3 - Q16  I try not to lift heavy things or push things around too much.” Step 3 - Q17  “it’s asking me if I worry about activities that involve my hand and yes I do. I will not do certain activities. I’ve stopped doing certain sports because of my arm.” Step 5 - Cog6  “So worrying that activities involved in everyday life even. That just repetitive motion in your arm, worrying that it might make things worse for lymphedema.” Step 5 - Cog7 |
|  | Impact of heat (eg, hot weather, hot water) | “If I had a hot bath, my arm would swell a little bit. So I avoid really hot water. I don’t even have a hot shower.” Step 3 - Q18  “Well, the summertime, I tend to stay indoors because of the humidity causes it to expand more and causes problems” Step 3 - Q19  “Heat is a big thing. Going away, hot summers, things like that. That concerns me.” Step 4 - FG3  “…in the summertime I am very conscious of heat and I am very conscious of hot water in a bath or a shower, because that does for me affect my arm.” Step 5 - Cog5  “And this is something that I worry about all the time, always. From the shower to the outside. What temperature do I have the HVAC set at. Everything. Because I do find that my lymphedema is impacted by temperature.” Step 5 - Cog7 |
|  | Treatment/caring for arm | “I have that fear that it’s going to get worse if I don’t keep up with this therapy and maintenance.” Step 1 - Q1  “I’m concerned about how long can you go without having that [massage therapy]?” Step 3 - Q24  “Knowing that I have that appointment every two weeks to help me stay on track has really helped me emotionally, because I know it’s there. It’s like that safety blanket for me.” Step 3 - Q27  “I don’t know if that affects my arm. I am not an expert in lymphedema. So a lot of things I do [self-care] because I have anxiety….a lot of anxieties I have is just because I don’t know.” Step 3 - Q25  “I do worry when I don’t go to massage.” Step 5 - Cog1  “I worry about not doing things [treatment/self care] and when I do things, I worry that they are not going to work.” Step 5 - Cog7 |
|  | Impact on others | “It’s a burden sometimes. I can’t do like lift certain things when I want to. I have to get my son to do it for me.” Step 3 - Q19  “I mean he [spouse] has always been really good about it [doing chores], but I feel that he does more than I do now and that bothers me. Step 3 - Q22  “It [lymphedema] impacted on them [family] and it caused a lot of worry in them. Step 3 - Q23  “I do worry that it [lymphedema] inconveniences and may be a burden on them [family].” Step 5 - Cog4 |
|  | Impact on social life | “It's annoying. I won't leave the house sometimes. It's just too much.” Step 1 - Q14  “Not going out.” Step 2 - Cog5  “And basically, it’s hard on…you can’t really participate in a lot of activities like you used to.” Step 3 - Q24  “Dating life? I’m a chick with one fat arm. Yeah. It’s horrible.” Step 5 - Cog1  “I wouldn’t participate in certain things because my arm was so big and I thought it was ugly.” Step 5 - Cog5 |
|  | Impact on mental health | “And then when it flares, you try to keep on a happy face, but in the back of your mind it’s like, oh my God, is this ever going to get better or is this now the new normal?...That’s very depressing…it puts me in a really bad place.” Step 1 - Q13  “I don’t know if that affects my arm. I am not an expert in lymphedema…a lot of anxieties I have is just because I don’t know.” Step 3 – Q25  “Well, you constantly, it’s constantly on your mind that you have to deal with this thing.” Step 3 - Q24  “You feel horrible all the time. You are always thinking about it.” Step 4 - FG3  “…the worry about my arm significantly impacts my ability to function mentally and….especially if I am feeling really preoccupied about my arm.” Step 5 - Cog4 |
|  | Body image | “Embarrassed, definitely.” Step 2 - Cog 1  “I don’t feel attractive at all.” Step 4 - FG3  “And then of course there is the physical disfigurement. I feel very disfigured by it.” Step 4 - FG3  “I’m particularly self-conscious when I’m meeting new people and you put out your hand and they realize you’ve got this weird paw that it looks like. So I feel super self-conscious.” Step 4 - FG3  “It [lymphedema] affects how I look. I look fat and ugly. I hate it.” Step 5 - Cog1  “I feel that my arm is very….there is a lot of atrophy in that arm now, so that arm is very flabby. And sometimes it looks almost purple…I’m just a bit self-conscious about it.” Step 5 - Cog2  “…a lot of times I would never have my arm showing. I couldn’t wear certain things. I wouldn’t participate in certain things because my arm was so big and I thought it was ugly. And then I thought I was ugly and there is a whole body image…” Step 5 - Cog5 |
|  | Clothing | “I would have to buy 2 or 3 sizes larger than would fit me and have everything altered.” Step 4 - FG1  “But it was still quite swollen to the point where I was having a hard time finding clothes that would fit.” Step 4 - FG1  “…we need coats in the winter, and it was very difficult to find some clothing that fit.” Step 4 - FG1  “Not wearing... there were many things I couldn't wear because my arm wouldn't fit into it.” Step 5 - Cog 5 |

*Denotes the step (1 – 5), type of interview (qualitative, cognitive, focus group) and patient study ID# or Focus group#; Step 1 – Concept Elicitation 1;

Step 2 – Pilot Testing 1; Step 3 – Concept Elicitation 2; Step 4 – Focus Groups; Step 5 – Pilot testing 2; Q – qualitative interview; Cog – cognitive interview;

FG – focus group
